# Quality Assessment of Chilled and Frozen Fish—Mini Review

**DOI:** 10.3390/foods9121739

**Published:** 2020-11-25

**Authors:** Ana M. Duarte, Frederica Silva, Filipa R. Pinto, Sónia Barroso, Maria Manuel Gil

**Affiliations:** 1 MARE—Marine and Environmental Sciences Centre, Polytechnic of Leiria, Cetemares, 2520-620 Peniche, Portugal; ana.c.duarte@ipleiria.pt (A.M.D.); frederica.g.silva@ipleiria.pt (F.S.); filipa.gomes@ipleiria.pt (F.R.P.); sonia.barroso@ipleiria.pt (S.B.); 2 MARE—Marine and Environmental Sciences Centre, Faculty of Science, University of Lisbon, Campo Grande, 1749-016 Lisbon, Portugal; 3 MARE—Marine and Environmental Sciences Centre, ESTM, Polytechnic of Leiria, Cetemares, 2520-620 Peniche, Portugal

**Keywords:** fish quality, food safety, chill, freeze, shelf-life

## Abstract

Fish is a very perishable food and therefore several storage strategies need to be employed to increase its shelf-life, guaranteeing its safety and quality from catch to consumption. Despite the advances in modern fish storage technologies, chilling and freezing are still the most common preservation methods used onboard. The present review aims to summarize strategies to increase the shelf-life of fresh (chilled) and frozen fish, as whole, gutted, or fillet, involving the assessment of different traditional cooling and freezing conditions of different fish species caught in different locations. Although there are other factors that influence the fish shelf-life, such as the fish species and the stress suffered during catch, storage time and temperature and the amount of ice are some of the most important. In addition, the way that fish is stored (whole, fillet, or gutted) also contributes to the final quality of the product. In most studies, whole chilled and frozen fish present longer shelf-life than those preserved as gutted and filleted. However, it should be noted that other factors related to the organism, capture method, and transport to the preparation/processing industry should be considered for shelf-life extension.

## 1. Introduction

Fish is a very perishable food being highly susceptible to oxidation and microbiological deterioration. Therefore, efficient storage strategies need to be employed in order to increase its shelf-life and guarantee its safety and quality from catch to consumption. The shelf-life of fish is dependent of several factors such as storage time, temperature, fish species, the stress suffered during catch, and the amount of ice [1].

Recent advances in modern fish storage technologies include high pressure processing, irradiation, pulsed light technology, pulsed electric field, microwave processing, radio frequency, and ultrasound [2]. Despite these emerging technologies with application in fish processing, fish chilling and freezing remain the most widely used preservation methods on board. These are methods for maintaining fish quality through low temperatures, which include refrigerated storage between 0 °C to 4 °C or frozen storage at −18 °C to −40 °C [3]. Fish chilling can involve the application of superchilling technology, which allows products to be less available for deteriorative processes, due to the freezing of the minor part of the product’s water content (5–30%) [3]. This method allows the avoidance of using external ice around the product, with consequent reduction of transport weight and cost, as well as longer shelf-life than chilled foods due to the reduction of the microbial activity [3]. Yet, autolytic, enzymatic, or other chemical reactions can be accelerated by this process [3]. On the other hand, fish freezing can be applied when the fish is in the pre-rigor mortis stage, allowing its greater quality when compared with the rigor mortis and post-rigor mortis stage [4]. Therefore, these preservation methods need to be optimized to increase fish shelf-life to guarantee its quality and safety, with consequent satisfaction of consumers requirements, reduction of economic losses from fishing industries and food waste. This optimization can involve the effect of freeze/chill temperature and time [5,6], thawing [7], fish preparation (i.e., whole and gutted) [8], and bleeding conditions [9].

The present review aims to summarize strategies to increase the shelf-life of fresh and frozen fish, involving different fish species and catch locations. Data from the last two decades was analyzed since there is little information from more recent years. Therefore, the studies included in the review are those that have focused on traditional chilling (in ice and/or refrigerator) and freezing methods for pelagic, demersal and benthopelagic fish species as whole, gutted and filleted, with special focus on storage temperature and time as factors that influence their shelf-life. Additionally, studies involving traditional sensory, physicochemical, biochemical, and microbiological methods for fish quality assessment were also included. The first two sections revise the pathways of fish degradation and the most common methodologies for the evaluation of fish quality. The last two sections describe several studies involving strategies for shelf-life increase of chilled and frozen fish.

## 2. Fish Freshness Degradation

Freshness is an attribute that refers to unfrozen fish when it maintains its sensory, chemical, and nutritional characteristics since its capture.

The freshness loss as a consequence of fish degradation process, begins immediately after capture (postmortem alterations), justifying the importance of their careful handling from capture to processing/commercialization to maintain the quality of this product. Such care will determine the enzymatic, bacterial, and oxidative activity whose speed and, consequently, the degradation process, depends on preservation methods applied, as well as fish species, size, capture method, temperature, storage type, and physical condition before death [10].

### 2.1. Postmortem Alterations

After the fish’s death, sensory, physicochemical and microbiological changes occur.

Sensory changes are related to the appearance, texture, odor and taste perceived by the senses. Such changes involve muscle browning or darkening by Maillard reactions (when subject to temperature rise) or by enzymatic activity, release of mucus consisting especially of mucin (glycoprotein which is an excellent substrate for bacterial development) with the release of an offensive odor [11]. Additionally, sensory changes also include muscular withdrawal or gaping and burst bellies (also called belly bursting or burnt belly), caused by the action of digestive enzymes present in the fish gut [12,13].

Chemical changes are detected by chemical analysis, to identify degradation level and compounds formation, to infer about the quality of the fish. On the other hand, physical changes allow to determine other degradation parameters, such as the assessment of tissues electrical resistance and muscle rigidity, that gradually decreases until the advanced state of degradation of the fish [11]. Both change types result from phenomena that interfere with pH value, nucleotide catabolism (rigor mortis), protein degradation, free amino acids, lipids, and undesirable compounds production (biogenic amines and volatile nitrogen compounds) [11].

When the fish dies and is stored in ice for 5 to 6 days, the microorganisms that are present are in the latency phase (also called lag or delay) while adapting to the new environment (dead fish), through the adjustment of survival and growth mechanisms due the oxygen absence. After their adaptation to the new environment, microorganisms grow exponentially, with the beginning of the logarithmic phase (also called log), constituting the main reason for fish degradation after the sixth day in ice. In fact, the bacterial action is the main responsible for the fish deterioration, when compared with the autolytic action which, in turn, is responsible for the loss of quality. This fact is sustained with the autolysis beginning in the digestive tract, which can be removed by evisceration [11]. In fact, when evisceration is performed in proper hygiene conditions, the action of digestive enzymes and bacteria migration from the intestinal flora to fish meat, are prevented [14].

Thus, postmortem degradation or postmortem aging of fish can include three stages, namely: Rigor mortis, autolysis, and bacterial spoilage.

### 2.2. Rigor Mortis

**Figure 1:**
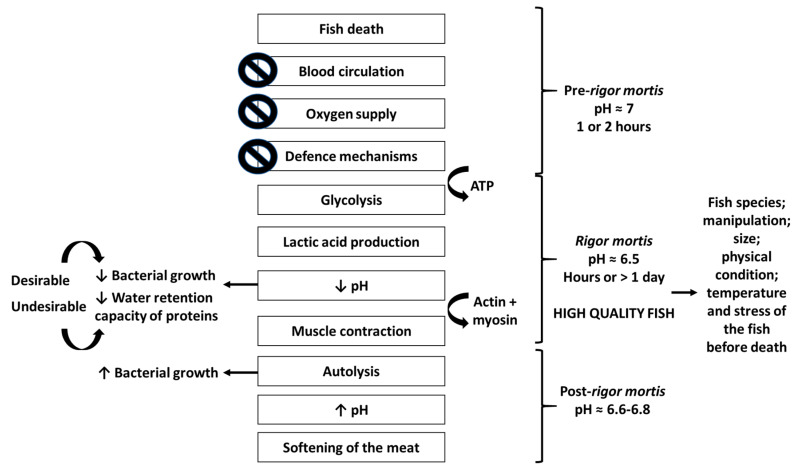
With the death of the fish, blood circulation, oxygen supply and defense mechanisms cease and pre-rigor mortis begins leading to glycolysis with expenditure of ATP (adenosine triphosphate) and lactic acid production. Consequently, the rigor mortis process begins when pH drops, reducing the microorganism’s growth (desirable effect) and the water retention capacity of proteins (undesirable effect). Actin and myosin bind, the muscle contracts and post-rigor mortis is initiated with the autolysis process. With the increase of microbial growth, the pH increases with the consequent softening of the fish meat. The post-rigor mortis starts after the end of the rigor mortis, which duration varies with fish species, manipulation, size, physical condition, temperature, and stress of the fish before death.

At the time of death, called pre-rigor mortis, the fish muscle contains glycogen, phosphocreatine and ATP (adenosine triphosphate), allowing its flexibility and elasticity for a few hours. With the fish’s dead, blood circulation and defense mechanisms are stopped, leading to the oxygen supply interruption with the beginning of glycogen anaerobic degradation, called glycolysis. Glycolysis will have an extension dependent on the living organism glycogen reserves, that are higher in well-fed fish and those with little agitated death, allowing the increase of fish shelf-life. This process causes the expenditure of ATP until very low values for the lactic acid production, with consequent pH decrease. Such decrease allows the reduction of bacterial growth (desirable effect) and water retention capacity of proteins (undesirable effect), with the beginning of rigor mortis phase [11]. In this phase, connections between contractile proteins (actin and myosin) are established, resulting in muscle contraction, becoming rigid and inextensible [12]. The rigor mortis resolution occurs after hours or more than a day, depending on the fish species, manipulation, size, physical condition and, mainly, the temperature and stress before death. Thus, with lower stress and temperature, the later it starts the longer the flesh stiffness will be maintained, being the fish before or during this phase synonymous of high quality. With the rigor mortis resolution, the autolytic processes are initiated and, consequently, the fish deterioration will begin due to the creation of a favorable environment to bacterial growth. As a consequence of the nitrogen compounds production, autolytic and bacterial reactions increase the pH as the preservation period increases [11]. Figure 1 schematizes the biological processes that are initiated at fish’s death leading to its degradation.

### 2.3. Autolysis and Bacterial Spoilage

Autolysis comprises the process of fish proteins and fats hydrolysis, by proteolytic and lipolytic enzymes action, respectively [10]. The responsible enzymes for the autolysis of proteins and collagen, with consequent softening of refrigerated fish muscle in the postmortem phase, are cathepsin, calpain, and collagenase [12]. In fact, at the early postmortem stage, changes in the fish texture are caused specially by lysosomal (cathepsins B and L) and cytosolic enzymes (calpain system), which cause the myosin heavy chain (MHC) hydrolysis [13,15]. This hydrolysis is mainly caused by cathepsins, while calpains are known to increase the proteases potential to hydrolyze myofibrillar proteins [13]. In addition, the degradation of collagen (type I and V), which increases the fish structure, also results in the softening of the muscle [13]. Other consequences of flesh autolysis involve the rupturing of cell walls and blooding, resulting in a water loss with both oil and protein present, contributing to the postmortem degradation of fish [16]. As a result, peptides and free amino acids can be produced promoting the microbial growth and production of biogenic amines, whose degradation rate depends on species and storage conditions [16]. However, at 0 °C there is a decrease in the reaction rates, giving rise to lipid autolysis and enzymatic action on lipids at freezing temperature (−18 °C), which helps to limit storage time in fatty fish [12].

### 2.4. Lipid Oxidation

The oxidation process involves oxygen and unsaturated lipids such as polyunsaturated fatty acids (PUFA), where a high degree of unsaturation results in greater susceptibility to oxidation, with consequent changes in taste and development of possible risks associated with the formation of peroxides [10]. Considering that the highest percentage of fat present in fish is constituted by unsaturated lipids, fatty fish, such as sardines (*Sardina pilchardus*) and horse mackerel (*Trachurus trachurus*), are most susceptible to oxidative processes [11]. The oxidation process begins with the formation of hydroperoxides, associated with color alterations in the fish tissue to brown or yellow, and subsequent degradation to aldehydes and ketones, resulting in a strong rancid taste. Additionally, oxidation can be initiated and accelerated by light, especially ultraviolet, as well as by organic and inorganic substances, such as copper and iron [11].

## 3. Evaluation of Fish Quality

The quality of fish is a term that includes not only its appearance and freshness, but is also related to food safety, such as the absence of harmful microorganisms or substances to consumer´s health. Thus, in general, fish quality involves the absence of microbiological and chemical risks and the maintenance of sensory, nutritional and physicochemical factors (e.g., humidity, pH, color, texture, and macronutrients) appropriate to its intended purpose.

The evaluation of fish quality comprises the combined use of different methodologies, due to the complexity of the decomposition process to which it is subjected. The most common are the sensory, physicochemical, biochemical and microbiological methods.

Sensory analysis is commonly defined as a “scientific method that evokes, measures, analyses and interprets people’s responses to products that are perceived through the five senses: Sight, odor, touch, taste, and hearing” [17]. One of the most used sensory methods in the evaluation of fish quality is the Quality Index Method (QIM). This method uses a category scale, where the scheme measures the changes in the degree and rate of important criteria, which can be converted into equivalent days of storage and remaining shelf-life [18]. In a meta-analysis developed with sixty-eight studies, the specie-specificity of QIM was assessed, due to some limitations such as no consideration of differences between species, mixture of subjective and objective sensory methods, the need for trained and experienced assessors and the absence of fish shelf-life information [19]. According to the authors, seafood group (bluefish, whitefish, Selachii, cephalopods, and crustaceans), storage procedure and temperature, family, and habitat, as well as maximum number of QIM demerit points have significant effects on correlation coefficients derived from QIM schemes [19]. However, the authors concluded that QIM’s categorization is not justified due to some moderate publication bias and influential analysis, thus the species-specificity of QIM schemes was not discarded [19]. QIM is applied to raw fish, while Torry scheme is used for freshness evaluation of cooked fish fillets to carry out their sensory evaluation [20]. Torry scheme can be applied to lean, medium fat fish and fatty fish using a 10-point scale where 10 refers to “very fresh in taste and odor”, 5.5 is the limit for consumption, 3 to “spoiled fish” and lowest scores are not applied since it is not adequate for human consumption [21]. Sensory descriptive tests, where it is possible to detect and describe sensory attributes perceived from a sample and also identify the nature of a sensory difference and/or assess the magnitude of that difference, are also commonly used in the evaluation of fish quality. For this purpose, a panel of at least 10–12 assessors are trained to be familiar with all sensory parameters applied to the product under study, its attributes and limits [22].

The physicochemical parameters applicable to fish are water activity (aw), color, texture, and pH.

The aw is the amount of available water for chemical reactions, enzymatic activity and microbial growth, with values close to 1 being associated to high perishability and values close to 0 being associated to low perishability. Thus, the determination of food’s aw is an important parameter in the evaluation of its deterioration susceptibility, in the prediction of its shelf-life and in the identification of its storage conditions for an extended shelf-life [23].

Color changes from autolytic and microbial activity in the fish degradation process may include the development of a yellowish color in the flesh or brown discoloration. Yellowish color in the flesh, occurs in some frozen fish as a result of chromatograph disruption with consequent release and migration to the subcutaneous layer, as well as due to lipid oxidation which also causes brown discoloration [24].

Fish texture is dependent on its fat and collagen contents and is a very important characteristic of fish muscle, which can be dry and hard in frozen products after thawing, revealing problems in freeze system and/or temperature maintenance [25]. Changes in fish texture can be assessed by light and electron microscopy, as well as by texturometers, being the latter more challenging in whole muscle due to the inherent nature of muscle tissues [20]. In addition, changes in protein size can be determined by electrophoretic and chromatographic techniques [20].

The pH determination throughout the fish storage, allows the identification of glycolysis phenomena through its reduction in postmortem fish, and subsequent increase to values above 7, due to the production of volatile compounds, which indicates advanced decomposition [26].

Besides the sensory and physicochemical methods, the quality of fish can also be accessed by biochemical methods such as thiobarbituric acid (TBA) and total volatile basic nitrogen (TVBN) determination.

Thiobarbituric acid or thiobarbituric acid reactive substances (TBARS) indicate the formation of secondary lipid oxidation products, such as the oxidation of peroxides into aldehydes and ketones and are frequently used to quantify the level of fish oxidation. The determination of the TBA index, is based on the extraction of the secondary malondialdehyde (MDA) compound, where a red complex is formed that is detectable by spectrophotometry [27].

During the fish storage, volatile compounds such as ammonia (NH_3_) and trimethylamine (TMA), are produced by autolytic and bacterial processes, resulting in an ammoniacal and strong fish odor, typical of deteriorated fish. TMA results from the bacterial reduction of trimethylamine oxide (TMAO), which occurs naturally in the marine organisms to allow osmotic regulation. The reduction of TMAO to TMA results from the bacterial action in the absence of oxygen and ice preservation. Additionally, TMA constitutes 1 to 5% (in dry weight) of the muscle tissue of fresh fish (varies according to species, catch area, size and physical condition), mainly from marine habitats. TMA is one of the main compounds assessed by total volatile nitrogen (TVBN), together with ammonia and other volatile amines [12,28]. The determination of TVBN to assess the fish freshness, considers all volatile nitrogen compounds in the sample, namely the levels of NH_3_, TMA and dimethylamine acid (DMA) that increase throughout the deterioration process [29]. However, due to the need of expensive laboratory equipment and trained operators, this time-consuming analysis is used in research but not often applied in fish industry [20].

Fish is the main cause of food-borne diseases, especially due to contamination by biological hazards (pathogenic bacteria, viruses, parasites, and biotoxins) whose occurrence are mainly due to improper handling practices, insufficient thermal treatments; inadequate chilling/cooling, absence of hygienic standards, cross contamination between raw and ready-to-eat foods, raw foods and contaminated ingredients, and inadequate cleaning of equipment and utensils [11].

The microorganism’s growth is affected by intrinsic and extrinsic factors. The intrinsic factors include substrate/fish limitations (pH, aw, oxidation-reduction potential, nutrients, antimicrobial constituents, and biological structures), while environmental limitations belong to the extrinsic factors (temperature, relative humidity, atmosphere, and external microbial activity). Among the extrinsic factors, the temperature that allows microorganisms growth and development stands out [14]. Total viable counts (TVC) is often used to assess the fish freshness, where 10^2^–10^6^ colonies forming units (cfu)/g are usual for whole and cut fish fillets, while 107–108 cfu/g are typically associated with sensory rejection [19]. Additionally, due to the low temperatures of chill storage, psychrotolerant microorganisms are favored and so their counting is a suggested measure of chill fish quality [20]. *Shewanella putrefaciens* is a typical deterioration bacteria of temperate water fish, conserved in an aerobic refrigerated environment. *S. putrefaciens* produces TMA, hydrogen sulfide (H_2_S), as well as other volatile sulfides, that are responsible for the smells and flavors of fish. Similar metabolites are produced by *Vibrionaceae* and *Enterobacteriaceae* bacteria during spoilage at high temperatures. On the other hand, some freshwater fish and many tropical water species, during ice storage and under aerobic conditions, are characterized by *Pseudomonas* deterioration associated with fruity and cloying odors. *Pseudomonas* produce different volatile sulfides (e.g., methylmercaptan CH_3_SH) and dimethyl sulfide ((CH_3_)_2_S), ketones, esters and aldehydes. Putrefaction or deterioration occurs very rapidly if the load of specific spoilage organisms exceeds approximately 107 cfu/g [11].

Besides the aforementioned methods, there are more recent techniques to assess fish quality and freshness, which are an alternative to traditional analysis with several benefits, such as rapid detection, objectivity, reliability, easy use, and minimal or no sample preparation [30,31]. These technologies include enzyme biosensor, electrochemical biosensor, electronic nose and tongue, colorimetric sensor, computer vision techniques, visible/near-infrared (Vis/NIR) spectroscopy, hyperspectral imaging (HSI) spectroscopy, fluorescence spectroscopy mid-infrared (MIR), near-infrared (NIR), and nuclear magnetic resonance (NMR) [30,31]. Regardless of these modern methods, it should be considered that their requirements regarding time, materials, equipment, and trained operators may not be suitable for industrial environment [32]. Therefore, there is a need to develop new equipment that allow the measurement of different attributes for industrial application, to assess the quality of the fish in a rapid, economic, and reliable way [32].

## 4. Deterioration of Fish Stored on Ice

When preserved on ice, the fish presents a typical pattern of deterioration divided into four stages, where the first two phases correspond to quality loss by autolytic processes, while the last two are associated with fish deterioration by bacterial action. The first stage is characterized by the high fish freshness, with sea smell (wild species) and a sweet taste. In the second stage, taste and odor are lost, without the development of unpleasant taste and maintenance of a pleasant texture. Deterioration cues such as off-odors, depending on fish species and metabolism type (aerobic/anaerobic), usually begins to occur in the third stage, with the development of “fishy smell”, ammonia and some sulfuric compounds. At the beginning of the third stage, the flavor may be slightly vinegary, fruity, or slightly bitter, especially in fatty fish, becoming, over time, an ammoniacal and sulfurous flavor with rancid smell development. The texture may be soft and watery or dry and hard. In the fourth phase, the fish is classified as deteriorated and putrid. Thus, fresh fishery products, with the exception of those kept alive, must be chilled as quickly as possible, and the time from catching to chilling should be limited to a maximum of 3 h and kept at a temperature close to that of melting ice, considering the conditions of survival of microorganisms [12].

Table 1 summarizes the changes that occurred in different fish species caught from different locations during refrigerated storage.

Sardines (*Sardina pilchardus*) captured in Morocco were subjected to sensory analysis, pH determination, determination of histamine content, and bacterial count for 18 days, being kept on ice at 2–4 °C, after 6 h of their capture. The results showed that after 9 days, according to the sensory and microbiological analysis, the product lacked quality. The product’s pH rises from 5.83 to 6.36, with a further rise after 18 days of storage. Through colorimetric and fluorometric methods, it was found that histamine concentration increased from 1.12 to 20 mg/100 g after 18 days, which can be possibly correlated with the pH rise [33]. Another investigation aimed to assess the sensory changes, the moisture content and the TBA index of sardines captured on the Galician Atlantic coast. The analyses started after 10 h of fish’s capture that was maintained in flake ice in the ratio of 1:1 (*w*/*w*) at 2 °C. There was a loss of sardine’s quality after 8 days, a difference in fresh and dehydrated mass from 71 to 73.5% after 19 days, as well as an increase in the index of TBA from 0.65 to 2.66 [34].

The importance of the fish’s storage temperature can be corroborated by Aubourg and Panguila studies on horse mackerel (*Trachurus trachurus*), where the first study achieved 14 days of fish shelf-life when kept at 0 °C, while the second author reported quality maintenance for 7 days when kept at 5 °C, both on ice [35,36].

Besides the storage temperature influence, the use of ice or refrigerator is also important as noted in Chudasama and colleagues work with Indian mackerel (*Rastrelliger kanagurta*). The authors evaluate the fish quality changes in both storage conditions, based on biochemical characteristics (TVBN and TMA) and sensory analysis. The results showed that fish stored in ice presented a more significant increase of TVBN, TMA, and pH values when compared with refrigerated fish. The sensory analysis reveals that after 5 days of ice storage the product shows degraded quality, unlike the refrigerated fish who maintain is quality after 7 days, possibly due to the lower temperature fluctuation to which the fish is subjected when refrigerated, compared to iced fish. In addition, the sensory data is corroborated by TVBN and TMA values (30 to 35 mg TVBN and 10 to 15 mg TMA/100 g of fish muscle) that indicate fish spoilage [37].

This correlation between sensory analysis and TVBN values, was not reported in Vásquez-Sánchez and colleagues work, with values lower than 30 mg/100 g during the entire storage. However, a high correlation was reported between MDA concentration and gill odors, with slight off-odors and flavors, as well as texture changes (hardness, chewiness and adhesiveness) being perceived by the assessors in cooked tilapia (*Oreochromis niloticus*) fillets after 10 days. The authors assume that these correlations can result from the products generated during lipid oxidation, were texture changes may result from myofibrillar proteins degradation by the MDA reaction with amino acids. The authors also reported a positive correlation between pH and TVBN, TBARS, TVC, and PC, as well as with hardness, chewiness and adhesiveness [38]. Similar texture results were reported by Xu and colleagues’ study with turbot (*Psetta maxima*), where hardness decreased over the time of storage. The authors reported a pH levels decrease attributed to lactic acid production and liberation of inorganic phosphate by ATP degradation, which was followed by a pH increase due to alkaline compounds accumulation [39].

Rong and colleagues evaluated the effect of gutting on Pacific saury (*Cololabis saira*) shelf-life during refrigerated storage. The authors reported that gutting slightly increases the initial microbial load due to bigger fish flesh surface exposure to the environment and gutting procedures (processing tables and knives). *Pseudomonadaceae* were the dominant bacteria in both fish samples, with lower initial levels were being reported in gutted fish with rapid increase through the remaining time of analysis. Regardless of the lower initial levels reported in gutted fish, the authors reported that this process accelerates *Pseudomonadaceae* growth and changes the fish microbiota. According to the authors, 7 log_10_ cfu/g, which is considered the normal acceptability level, was almost reached after 10 and 6 days for whole and gutted fish, respectively, as well as TVBN values close to 30 mg/100 g. In addition, a TBARS value of 5 mg/kg, which is regarded as the spoilage level of fatty fish, was almost reached at day 10 for gutted fish and surpassed after 14 days in whole fish [8].

### Refrigeration Temperature Requirements

The maintenance of quality through careful handling involves immediate cooling at the time of capture, avoidance of temperature abuses and a high cleanliness degree on the boat’s cover and hold. On board, cooling is the most critical operation in the fish handling process, generally using ice obtained from drinking water in a given proportion, to keep the fish fresh as close to the freezing point as possible (0 °C) [10]. The refrigeration on industry environment, should be carried out with cool water at 0 to 3 °C, while fish thermal center (spine) must be maintained at temperatures between 0 and 2 °C.

## 5. Deterioration of Frozen Fish

The deterioration of frozen fish depends on the freezing rate, storage temperature and oxygen, temperature fluctuations and transport stages. Freezing is based on the ice crystals formation, which are larger in slow process, causing protein denaturation, cell membranes rupture with fluids loss on thawing, which results in a low-quality product. When the freezing process is performed quickly, the crystals are smaller, minimizing fluid losses on defrosting, resulting in a high-quality product. However, temperature fluctuations induce recrystallization, reducing the product’s quality that becomes equivalent to a slow freezing product [12,40]. Therefore, changes in frozen fish quality involve change color (due to deterioration at the food surface, chemical, and biological actions), weight loss (both induced by ice crystal growth), increased enzymatic activity and lipid oxidation [40]. The textural changes in frozen fish do not have a fully known mechanism, but it is believed that they are essentially due to the transformation on myofibrils water retention capacity, with formaldehyde and DMA formation. Such capacity is altered by the spacing/compression between fibers by the ice formed between myofibrils or by certain transformations that make the muscle fibers unable to absorb the water lost in their freezing, preventing them from recovering volume [12,40].

Table 2 summarizes some studies that have been conducted in order to evaluate fish changes through the freezing process.

Aubourg and colleagues conducted a study with horse mackerel captured in Spain that was kept in freezer at −80 °C and −20 °C, after 10 h of capture, and sensory analyses determined after 12 and 5 months of shelf-life, respectively. For both temperatures, moisture content varied between 74 and 78%. TBA content increased up to 5 months of storage at both temperatures tested, followed by a reduction after one year [41]. In a previous study of the same authors, 5 months of shelf-life are achieved when horse mackerel is first kept on ice (0–2 °C) for 5 days, followed by freezing at −80 °C and then at −20 °C. The same study confirmed that when the initial refrigeration is carried out for 1 to 3 days, the shelf-life increases to 7 months [42]. In addition, unlike what was reported in the latest study of Aubourg and colleagues, TBA values were higher in the final months of the study [42].

This increase in TBA values was also reported by Calanche and colleagues, that studied whole, gutted and filleted seabream [43]. The authors concluded that filleted fish has lower shelf-life, higher microbial load, and higher free amino acids content than gutted and whole fish. The presence of hydroxyproline was associated with whole fish, arginine and glutamine with gutted fish and glycine, taurine and glutamic acid with fillets [43]. However, higher microbial loads are reported on fish skin when compared with fillets and especially with fish muscle, according to Popelka and colleagues in rainbow trout [44]. The authors performed the microbiological analysis on fresh fish samples on its arrival to laboratory and after 7 days, and 1, 3, and 6 months frozen. Those microbiological analysis involve TVC, PC, and *Pseudomonas* detection. The TVC and PC revealed a slight colony count decrease between fresh fish (muscle and fillet) and frozen fish (after 1 month), while no count changes were observed on the skin. On TVC experiment, a higher increase between the third month frozen and the sixth month was reported for all the samples. On the other hand, PC experiment reveal that the highest content of *Pseudomonas* was reported for skin and fillet samples, being absent during the storage of frozen muscle samples. In addition, pH values did not present statistical differences in frozen samples, while TVBN values from fresh samples were significantly different from the frozen ones [44].

Despite the difference between species, the increase of fillets shelf-life may be achieved through dark muscle removal by deep skinning, according to a study with herring by Dang and colleagues [45]. The authors revealed that the dark muscle is more sensitive to lipid oxidation than the light muscle, after having analyzed herring fillets for 14 months at stable temperature (between −12 °C and −10 °C) and stress conditions [45]. The stability of fish subjected to temperature abuse, was also a case of study in Romotowska and colleagues work with mackerel [46]. In their investigation, two types of samples were analyzed: One subjected to a temperature abuse (−12 °C) for one month followed by storage at constant temperature (−25 °C) for 9 months and the other with stable storage (−25 °C), in both cases with samples collected in July and September. The results revealed no statistical differences between conditions regarding water and lipid content, as well as an increase in lipid oxidation and free fatty acids content, with consequent effect on the quality and stability of the product. In addition, according to the authors, the free fatty acids content showed a possible increase of enzymatic activity due to temperature fluctuation, as lipid deterioration cannot be inhibited in proper conditions after damage has been done [46].

The quality of frozen products is closely related with the freezing settings, but also with thawing conditions due to their influence on chemical reactions and muscle degradation. To test the influence of thawing temperature media in the quality of Atlantic cod fillets, two different temperatures were applied in an air circulation system [7]. One batch was thawed at 10 °C for 4 h, the other was thawed at the same temperature for 2 h and lowered to −0.5 °C for 26–27 h and them both batches were filleted and kept at 2.9 ± 0.6 °C for 6 days. The results showed no statistical differences on sensory evaluation over the time of analysis, with low prevalence of muscle redness and blood spots. This was an indication of the good quality of fillets from both batches, as well as gentle capture with little bruising along with sufficient bleeding after capture. In addition, no statistical differences were reported in fillets texture, TVC, H_2_S-producing bacteria and TVBN. That lack of differences between thawing methods and detection of shelf-life limit may be explained, according to the authors, with the short time of analysis [7].

The slow thawing effect was studied in other investigation, where the evolution of pH, amino nitrogen and nitrogen from amino acids were assessed in carp (*C. carpio*), catfish (*S. glanis*), mackerel (*S. japonicus*) and hake (*M. merluccius*) for 48 h with sampling at time 0 and after 3, 9, 15, 21, 30, 36, 42, and 48 h from thawing [47]. At time 0, small or non-significant differences were observed between the species. After 9 and 30 h, all the species revealed an increased pH value, being the 30 h also marked by the maximum level of nitrogen from amino acids (NAA). Between 9 and 15 h the largest increase of TMA values was reported for all the species, with the exception of carp (42 to 48 h), the highest values were reported in catfish and the lowest in mackerel. According to the authors, 0–1 mg/100 g TMA indicates fresh fish, 1–5 mg/100 g relatively fresh fish and 5 mg/100 g altered fish, which are not concordant with those previously reported by Chudasama and colleagues where 10–15 mg/100 g indicates fish spoilage [37,47]. Such differences will affect the judgment about the fish freshness and, consequently, its shelf-life. However, it should be noticed that there are different legal limits in each country (e.g., 25–35 mg/100 g according with European regulations), and different limits are established for each experiment, which can influence the range values adopted by each author. Thus, according to Avramiuc, after 9 h of thawing only mackerel remained relatively fresh and after 15 h all the samples were spoiled [47]. According to the author, catfish and hake had the highest spoilage speed and mackerel the lowest one.

Nevertheless, in Hematyar and colleagues’ study with carp, a shelf-life of 24 weeks was achieved through a freezing storage of carp fillets at −20 °C [48]. However, in their study the thawing process was not a case of study. In their investigation, it was found that the firmness of frozen carp fillets decreased due to the freezing process but not due to the storage time. In addition, the authors also reported a correlation between lipid oxidation and sensory aspects [48].

Besides the temperature fluctuation, blood and blood components, like myoglobin and hemoglobin, in the fish muscle also contribute to lipid oxidation, which varies between species and depends on the bleeding conditions (e.g., bleeding time, medium, and temperature). Phospholipids are the main components of blood lipids and are highly susceptible to oxidation, suggesting that blood lipids contribute to lipid oxidation [9]. As a result, Nguyen and Phan, investigated the effect of bleeding conditions on the quality and lipid degradation of farmed cobia (*Rachycentron canadum*) fillets during storage. For that purpose, the authors studied three test groups in: (1) Unbleeding fillets; (2) cut in the throat and bled in the air for 15 min; (3) cut in the throat and bled in iced water (4 ± 1 °C) for 15 min followed by air packaged in polyethylene bags, air blast freeze (−35 °C) for 3 h and stored in carton boxes (−20 ± 2 °C) for 24 weeks. The results showed that the ice water bleeding favors lower heme and non-heme iron contents in the fish muscle. In addition, it was confirmed that lipid degradation had high correlations with heme and non-heme iron content, and that the lipid oxidation and heme pigments are the main cause for flesh discoloration during frozen storage [9].

### Freezing Temperature Requirements

Due to the importance of maintaining the time-temperature binomial in the quality of frozen and chilled products, the time-temperature tolerance theory was developed. This theory states that: For each frozen product, there is a relationship between storage temperature and the time it takes to change the product’s quality; changes during storage and distribution at different temperatures are cumulative and irreversible over the entire storage period [49].

As the temperature below 0 °C is the critical zone of deterioration by protein denaturation, in rapid freezing it is recommended that the temperature of all fish should be reduced from 0 °C to −5 °C in 2 h or less. The temperature must then be reduced again until an average storage temperature of −30 °C is obtained at the end of the freezing process. The latter requirement is that the warmest part of the fish (spine) is reduced to −20 °C at the end of the process. When this temperature is reached, the coldest parts of the fish will be at the freeze equipment temperature or close to −35 °C, while the average temperature will be close to −30 °C [49]. This is one of the quick freeze definitions, that ensures a good quality product.

## 6. Conclusions

The fish degradation is initiated in its capture, making this a crucial step to determine the quality and safety of this food product. Subsequently, the refrigerated and/or frozen storage conditions, also play a decisive role in the type of product that will be marketed and later consumed by the customer. In fact, if such conditions are optimized, the desired safety parameters will be achieved, as well as those of quality which, despite differing between consumers, present some common points that must be respected. Thus, it will be possible to sell conform products that satisfy the customer needs, assist in the economy of the fishing industries and fishermen, as well as to avoid food waste by extending the fish shelf-life.

The optimization of cooling and freezing conditions differs, among other factors, with the fish species, as well as with the form the fish is conserved to be sold (fillet or whole). Regarding refrigeration conditions, as a general rule, studies indicate that storage on ice at 0 °C allows a shelf-life of 14 days, which is reduced to 8 days when the ice:fish ratio is 1:1 (*w*/*w*) at 2 °C and 10 days when kept between 0–1 °C in plastic bags in the same proportion. In addition, the shelf-life is reduced to 5 days when the fish is kept in polystyrene and ice boxes at 2–4 °C, for 7 days at 5 °C with the same storage system, this time also being reached when the product is kept refrigerated at 2–4 °C. Thus, it is possible to conclude that the storage temperature is essential to determine the fish shelf-life, as well as the way it is preserved. In most studies whole fish had longer shelf times compared to gutted fish. The same effect of temperature has been reported by studies with frozen fish, where filleted products maintain sensory quality for up to 11 days, whereas whole and gutted fish are considered acceptable between 11 to 18 days, when stored for 1 month at −30 °C. However, it was also found that filleted fish, kept in plastic bags at −20 °C has a shelf-life of 24 months. Thus, it should be noted that fish’s shelf-life, as mentioned above, depends on several factors related to the organism (species, habitat, food, etc.), with method of capture, transport to the preparation/processing industry, that must be also considered when optimizing fish freezing and refrigeration conditions. This review is expected to be helpful to elucidate the most adequate storage conditions for a given fish species.

## Figures and Tables

**Table 1:** Sensory, physicochemical, biochemical and microbiological changes of refrigerated fish recorded in the literature.

Fish Species	Origin	Storage Conditions	Type of Analysis and Results							Reference	
			Sensory	Physicochemical			Biochemical		Microbiological		
Sardine (*Sardina pilchardus*)	Morocco (analyzed 4–6 h after capture)	Layers of sardines and ice in polystyrene boxes (2–4 °C)	Shelf-life: 9 days Hardly bright, rigor mortis moderately tense, cloudy eyes, absent gills cover, scales fall easily, anus partially open	pH	Day 0: 5.83		-		TVC ^1^ (cfu/g): 10^6^–10^7^ on 9th day	[33]	
					Day 9: 6.36						
					Day 18: 6.57						
	Atlantic Coast (analyzed 10 h after capture)	Flake ice 1:1 (*w*/*w*) (2 °C)	Shelf-life: 8 days Limiting factors: Gills and flesh odor	Moisture (%): 71–73.5			TBA ^2^ (mg MDA ^3^/kg)	Day 0: 0.65	-	[34]	
								Day 19: 2.66			
Horse mackerel (*Trachurus trachurus*)	Unknown (analyzed 10 h after capture)	Ice (0 °C)	Shelf-life: 12 days classified as “2—fair quality” in a scale from 1 (rejectable) to 4 (highest quality)	Moisture (%): 75–80			TBA ^2^ (mg MDA ^3^/kg)	Day 0: 0.10	-	[35]	
								Day 19: 0.85			
Horse mackerel (*Trachurus trachurus*)	Portugal	Covered with ice in polystyrene boxes (5 °C)	Shelf-life: 7 days by QIM ^4^ demerit points variation: 13.40 ± 1.92 (Portugal) and 11.25 ± 1.75 (Luanda) Limiting factors: Color, gills odor and muscle firmness	Moisture (%): 71–77			TBVN ^5^ (mg/100 g)	Day 0: 12.37 ± 0.802	-	[36]	
				Color:	Day 0: L*^6^ = 44.79 ± 11.74; a*^6^ = −1.63 ± 0.44; b*^6^ = −1.63 ± 1.09						
					Day 7: L*^6^ = 60.85 ± 16.53; a*^6^ = 0.32 ± 2.2; b*^6^ = 1.92 ± 5.04			Day 7: 46.69 ± 0.34			
				aw: 0.98							
				pH	Day 0: 6.34 ± 0.18		TBA (µg MDA/g)	Day 0: 1.47 ± 0.00			
					Day 7: 6.33 ± 0.057						
				Texture (hardness) (kgf)	Day 0: 525.01 ± 81.797			Day 7: 11.50 ± 2.18			
					Day 7: 474.31 ± 102.72						
	Luanda			Moisture (%): 73			TBVN ^5^ (mg/100 g)	Day 0: 10.86 ± 0.54			
								Day 7: 30.15 ± 3.45			
				pH	Day 0: 6.32 ± 0.10		TBA (µg MDA/g)	Day 0: 1.80 ± 0.58			
					Day 7: 6.43 ± 0.112			Day 7: 7.44 ± 2.84			
Indian mackerel (*Rastrelliger kanagurta*)	Obtained from Indian fishing harbor	Ice (2–4 °C)	Spoiled: 5 days Appearance, color, odor and overall acceptability classified as 5—neither like or dislike	Moisture (%)	Day 0: 74.37 ± 0.19		TVBN (mg/100 g)	Day 0: 3.8 ± 0.18	-	[37]	
					Day 3: 77.72 ± 0.20						
					Day 7: 78.41 ± 0.12			Day 3: 22.4 ± 1.55			
				Total fat (%)	Day 0: 4.11 ± 0.12						
					Day 3: 3.95 ± 0.07			Day 7: 41.3 ± 1.47			
					Day 7: 4.18 ± 0.16						
				Crude protein (%)	Day 0: 18.46 ± 0.18		TMA ^7^ (mg/100 g)	Day 0: 1.11 ± 0.19			
					Day 3: 17.19 ± 0.13						
					Day 7: 16.79 ± 0.04			Day 3: 5.66 ± 0.22			
				pH	Day 0: 5.67 ± 0.15						
					Day 3: 6.50 ± 0.30			Day 7: 14.73 ± 0.15			
					Day 7: 7.27 ± 0.21						
		Refrigerator chilled (2–4 °C)	Good quality: 7 days (end of the study) Appearance, color, odor and overall acceptability classified as 6—like slightly	Moisture (%)	Day 0: 74.37 ± 0.19		TVBN (mg/100 g)	Day 0: 3.8 ± 0.18			
					Day 3: 73.39 ± 0.26			Day 3: 9.2 ± 0.35			
					Day 7: 72.35 ± 0.28			Day 7: 25.9 ± 0.61			
				Total fat (%)	Day 0: 4.11 ± 0.12		TMA ^7^ (mg/100 g)	Day 0: 1.11 ± 0.19			
					Day 3: 4.28 ± 0.03						
					Day 7: 4.33 ± 0.14						
				Crude protein (%)	Day 0: 18.46 ± 0.18			Day 3: 2.86 ± 0.25			
					Day 3: 17.90 ± 0.15						
					Day 7: 17.17 ± 0.22						
				pH	Day 0: 5.67 ± 0.15			Day 7: 9.80 ± 0.23			
					Day 3: 6.27 ± 0.25						
					Day 7: 6.53 ± 0.25						
Tilapias (*Oreochromis niloticus*)	Brazil	Layers of ice and fish 1:1 (w:w) (0–1 °C) in plastic trays and refrigerated (1 ± 0.5 °C)	Spoiled: 10 days Fishy odor, slightly sour flavor, trace of “off flavor” very dry and fibrous texture.	Color: Ventral and dorsal area redness decrease in the first 6 and 3 days, respectively; yellowness was negatively correlated with days of storage			TVBN (mg/100 g): Lower than 30 during the entire storage		TVC (ISO 4833–2:2013) (log cfu/g): 3.32 ± 0.05 at 13th day	[38]	
				Texture: Negative correlation between days of storage and the hardness and chewiness; adhesiveness was positively correlated with days of storage							
							TBARS (mg MDA/kg): Up to 0.474 after 13 days				
				pH: Do not exceeded 7 during the entire storage.					PC ^8^ (ISO 17410:2001) (log cfu/g): 3.59 ± 0.06 at 13th day		
Turbot (*Psetta maxima*)	China (local market)	Refrigerated (4 ± 0.5 °C, 67% relative humidity)	Shelf-life: 8 days by QIM acceptable appearance, gill, eye color and flesh odor. Limiting factors: Ammoniac odor, acidic taste and darker color.	pH: Initial decrease followed by increase			TVBN (mg/100 g)	Day 0: 6.36 ± 0.04	TVC (log cfu/g)	Day 0: 3.15	[39]
				Texture: Hardness, springiness and resilience decreased during the storage							
								Day 20: 44.34 ± 2.04		Day 20: 6.67	
				Color: Whiteness minimum value at day 0 and maximum on 20th day							
Pacific saury (*Cololabis saira*)									Mainly *Pseudomonadaceae*: 90% on day 8	[8]	
Pacific saury (*Cololabis saira*)	North Pacific Quick-freeze (−35 °C, less than 2 months) on the vessel	Transported at −28 °C, stored (2 ± 1 °C)	Gutted	Spoiled: 6–8 days Classified below 6—acceptable in a scale from 0 to 9 Limiting factors: Odor deterioration, and the secondary cause was the altered appearance		-	TVBN (mg/100 g): values close to 30 on day 6	TVC (Log_10_cfu/g)	Day 0: 3.75	[8]	
									Day 6: Close to 7		
							TBARS ^9^ (mg MDA/kg): values close to 5 on day 10	Mainly *Pseudomonadaceae*	Day 0: 35.8%		
									Day 8: 95%		
			Whole	Acceptable: 10 days Classified as 6—acceptable in a scale from 0 to 9		-	TVBN (mg/100 g): Values close to 30 on day 10	TVC (Log_10_cfu/g)	Day 0: 3.29		
							TBARS ^8^ (mg MDA/kg): Values close to 5 on day 14		Day 10: Close to 7		

^1^—Total viable count; ^2^—thiobarbituric acid; ^3^—malondialdehyde; ^4^—Quality Index Method; ^5^—total volatile base nitrogen; ^6^—L*: lightness, a*: red/green value, b*: blue/yellow value; ^7^—trimethylamine; ^8^—Psychrotrophic count; ^9^—TBA reactive substances.

**Table 2:** Sensory, physicochemical, biochemical, and microbiological changes of frozen fish recorded in the literature.

Fish Species	Origin	Storage Conditions		Type of Analysis and Results							Reference
				Sensory	Physicochemical		Biochemical		Microbiological		
Horse mackerel (*Trachurus trachurus*)	Spain (analyzed 10 h after capture)	Control sample (raw fish)		-	Moisture (difference between fresh and dehydrated mass): 74–78% (for all the samples)		TBA: 0.17 mg MDA/g		-		[41]
		Polyethylene bags	−80 °C	Shelf-life: 12 months. Classified as A—good quality in a scale from E (highest quality) to C (rejectable)			TBA (mg MDA/g)	1st month: 0.19	-		
								5th month: 0.72			
								12th month: 0.22			
			−20 °C	Shelf-life: 5 months. Classified as B—fair quality in a scale from E (highest quality) to C (rejectable)			TBA (mg MDA/g)	1st month: 0.26			
								5th month: 0.85			
								12th month: 0.75			
	Unknown (analyzed 10 h after capture)	Frozen at −80 °C and then kept at −20 °C for 0 to 7 months	Chilled on ice (0–2 °C) for 0 and 1 days	Shelf-life: 7 months. Classified with good quality (0–29); in a scale from 0 (no rancidity at all) to 100 (maximum rancidity).	Moisture (difference between fresh and dehydrated mass): 750–790 g/Kg (for all the samples)		TBA: Absence of values, but higher values were obtained after 5–7 months for products previously refrigerated for 3–5 days		-		[42]
			Chilled on ice (0–2 °C) for 3 and 5 days	Shelf-life: 5 months. Classified with fair quality (30–59).							
Seabream (*Sparus aurata*)	Spain (local farm)	Ice (0 ± 1 °C) in polyspan boxes for 5–18 days and 1 month frozen (−30 °C) in filleted form	Whole	Shelf-life: 11–18 days. Classified with 6.7 and 6.6, respectively, in Torry scheme from 8 (very fresh) to 1 (rotten)	Free amino acids: Whole and gutted fish were similar; filleted have the highest levels		TVBN (mg/100 g): Highest values on filleted fish at day 5, with significant increase over the time and highest values of all (86.2)	PC: Statistical differences between filleted and whole fish, where the last was similar to gutted. Filleted and gutted with the highest values.	[43]		
			Gutted	Shelf-life: 11–18 days. Classified with 7.8 and 4, respectively, in Torry scheme from 8 (very fresh) to 1 (rotten)							
							TBARS (mg MDA/kg): Highest values on filleted fish at day 5, with significant increase over the time and highest values of all (1.5)				
			Fillet	Shelf-life: <11 days. Classified with 6.6 and 6.7, respectively, in Torry scheme from 8 (very fresh) to 1 (rotten)							
Rainbow trout (*Oncorhynchus mykiss*)	Farmed in Slovakia (vacuum packed and cooled at 0–2 °C)	−18 °C		-	pH	Fresh: 6.51 ± 0.03	TVBN	Fresh: 12.75 ± 0.52	TVC (log cfu/g) Skin	Fresh: 4.75 ± 0.15	[44]
										1 month frozen: 4.75 ± 0.25	
										3 months frozen: 4.8 ± 0.2	
						1 month frozen: 6.79 ± 0.01		1 month frozen: 17.03 ± 0.1		6 months frozen: 7.05 ± 0.45	
									PC (log cfu/g) Skin	Fresh: 4.8 ± 0.1	
										1 month frozen: 4.8 ± 0.1	
						3 months frozen: 6.78 ± 0.05		3 months frozen: 17.03 ± 0.21		3 months frozen: 4.5 ± 0.6	
										6 months frozen: 4.9 ± 0.5	
									*Pseudomonas* detection (log cfu/g) Skin	Fresh: 1.5 ± 1.06	
						6 months frozen: 6.75 ± 0.02		6 months frozen: 18.74 ± 1.71		1 month frozen: 2.63 ± 0.08	
										3 months frozen: 2.65 ± 0.26	
										6 months frozen: 3.33 ± 0.37	
Rainbow trout (*Oncorhynchus mykiss*)	Farmed in Slovakia (vacuum packed and cooled at 0–2 °C)	−18 °C		-	-		-		TVC (log cfu/g) muscle	Fresh: 2.7 ± 0.2	[44]
										1 month frozen: 2.1 ± 0.4	
										3 months frozen: 2.2 ± 0.2	
										6 months frozen: 5.05 ± 0.36	
									PC (log cfu/g) muscle	Fresh: 2.75 ± 0.05	
										1 month frozen: 2.05 ± 0.05	
										3 months frozen: 1.9 ± 1.14	
										6 months frozen: 1.75 ± 0.45	
									*Pseudomonas* detection (log cfu/g) muscle	Fresh: 1.13 ± 1.41	
										1 month frozen: 0	
										3 months frozen: 0	
										6 months frozen: 0	
Rainbow trout (*Oncorhynchus mykiss*)	Farmed in Slovakia (vacuum packed and cooled at 0–2 °C)	−18 °C		-	-		-	TVC (log cfu/g) fillet	Fresh: 4.55 ± 0.35		[44]
									1 month frozen: 3.7 ± 0.3		
									3 months frozen: 4.1 ± 0.3		
									6 months frozen: 6.05 ± 0.55		
								PC (log cfu/g) fillet	Fresh: 4.55 ± 0.45		
									1 month frozen: 3.8 ± 0.2		
									3 months frozen: 4.1 ± 0.4		
									6 months frozen: 3.9 ± 0.1		
								*Pseudomonas* detection (log cfu/g) fillet	Fresh: 1.28 ± 1.68		
									1 month frozen: 2.53 ± 0.13		
									3 months frozen: 2.73 ± 0.38		
									6 months frozen: 2.85 ± 0.57		
Herring (*Clupea harengus*)	Southwest coast of Iceland (filleted on the vessel, −25 °C, 2 months)	Fresh		-	Moisture (%)	Dark muscle: 62.1 ± 0.2	-		-		[45]
						Light muscle: 72.2 ± 0.4					
		−12 °C, 1 month followed by −25 °C (stress condition)			Moisture (%)	No statistical differences in light muscle.	TBARS: Increase rapidly, maximum after 3.5 months of storage. No statistical differences in light muscle.				
					Total lipids (%)	Higher in dark muscle. No statistical differences in light muscle.					
					Phospholipids (%)	Lower values on both muscles					
					FFA ^2^ (%)	Higher values on both muscles					
		−25 °C (stable condition)			Moisture (%)	No statistical differences in light muscle. Higher in dark muscle.	TBARS: No statistical differences in light muscle.				
					Total lipids (%)	Higher in dark muscle (less than stress condition). No statistical differences in light muscle.					
					Phospholipids (%)	Higher values on both muscles					
					FFA ^2^ (%)	Lower values on both muscles					
Mackerel (*Scomber scombrus*)	South-East of Iceland	−12 °C, 1 month followed by −25 °C (stress condition) for 9 months		-	No significant differences in total lipid and water content between both tested groups		TBARS: Maximum value after 3 months		-		[46]
							Free fatty acids: Less prone to lipid hydrolysis				
		−25 °C (stable condition) for 9 months					TBARS: Steady increase in the whole storage				
							Free fatty acids: More prone to lipid hydrolysis				
Cod (*Gadus morhua*)	Norwegian sea headed and gutted on the vessel (−40 °C)	−28 °C, 9 weeks, thawed with air diffusion (10 °C, 4 h), filleted, kept at 2.9 ± 0.6 °C		Quality maintenance after 6 days by QIM classified with 1.3 ± 0.4 for texture (from 0—firm to 2—soft), 1.4 ± 0.5 for color (from 0—bright to 4—yellow mucous), 1.7 ± 0.7 for odor (from 0—fresh to 4—ammonia and off flavors)	pH	Day 0: 6.6 ± 0.2	TVBN (mg/100 g)	Day 0: 12.5 ± 2.3	TVC (log cfu/g)	Day 0: 2.8 ± 0.2	[7]
						Day 6: 6.7 ± 0.1					
					Moisture (%)	Day 0: 82.1 ± 0.1				Day 6: 4.8 ± 0.4	
						Day 6: 81.5 ± 0.2					
					Texture: No statistical differences in shear forces and softness			Day 6: 18.8 ± 12.9	H_2_S-producing bacteria (log cfu/g)	Day 0: not detected	
										Day 6: 2.3 ± 0.6	
		−28 °C, 9 weeks, thawed with air diffusion (10 °C, 2 h followed by −0.5 °C, 26–27 h), filleted, kept at 2.9 ± 0.6 °C		Quality maintenance after 6 days by QIM classified with 1.4 ± 0.4 for texture (from 0—firm to 2—soft), 1.4 ± 0.5 for color (from 0—bright to 4—yellow mucous), 1.9 ± 0.7 for odor (from 0—fresh to 4—ammonia and off flavors)			TVBN (mg/100 g)	Day 0: 12.6 ± 1.8	TVC (log cfu/g)	Day 0: 1.8 ± 0.4	
					pH	Day 0: 6.8 ± 0.2				Day 6: 4.2 ± 0.8	
						Day 6: 6.8 ± 0.2					
					Moisture (%)	Day 0: 81.8 ± 0.2		Day 6: 15.0 ± 5.2	H_2_S-producing bacteria (log cfu/g)	Day 0: not detected	
						Day 6: 81.3 ± 0.1				Day 6: 1	
Carp (*Cyprinus carpio*)	Romanian streams (whole fresh live)	Slow thawing at room temperature (20–22 °C), slaughtered, eviscerated and frozen (−29 °C), kept 2 months up to the experiment		-	pH	Time 0: 6.35 ± 0.78	TMA (mg%)	Time 0: 0.37 ± 0.02	-		[47]
								After 9 h: 1.53 ± 0.11			
						After 9 h: 6.65 ± 0.78		After 15 h: 4.68 ± 1.05			
								After 30 h: 8.53 ± 0.64			
						After 15 h: 6.83 ± 1.07		After 42 h: 10.33 ± 1.19			
						After 30 h: 7.35 ± 0.93		After 48 h: 13.75 ± 0.72			
						After 42 h: 7.58 ± 0.49	NAA ^1^ (g%)	Time 0: 0.06 ± 0.008			
								After 9 h: 0.09 ± 0.005			
						After 48 h: 7.66 ± 0.92		After 15 h: 0.18 ± 0.03			
								After 30 h: 0.31 ± 0.04			
								After 42 h: 0.23 ± 0.03			
								After 48 h: 0.17 ± 0.01			
Catfish (*Silurus glanis* L.)				-	pH	Time 0: 6.40 ± 0.91	TMA (mg%)	Time 0: 0.50 ± 0.09	-		
								After 9 h: 2.36 ± 0.07			
						After 9 h: 6.74 ± 0.63		After 15 h: 7.58 ± 1.37			
								After 30 h: 12.98 ± 1.88			
						After 15 h: 6.90 ± 0.48		After 42 h: 15.08 ± 1.08			
						After 30 h: 7.46 ± 0.59		After 48 h: 17.13 ± 1.92			
						After 42 h: 7.64 ± 1.23	NAA ^1^ (g%)	Time 0: 0.07 ± 0.005			
								After 9 h: 0.15 ± 0.04			
								After 15 h: 0.21 ± 0.09			
								After 30 h: 0.38 ± 0.09			
						After 48 h: 7.85 ± 0.38		After 42 h: 0.25 ± 0.06			
								After 48 h: 0.19 ± 0.09			
Mackerel (*Scomber japonicus* Houttuyn)	Retail (eviscerated frozen)	Slow thawing at room temperature (20–22 °C)		-	pH	Time 0: 6.38 ± 1.04	TMA (mg%)	Time 0: 0.73 ±0.07	-		[47]
								After 9 h: 0.98 ± 0.05			
						After 9 h: 6.65 ± 0.39		After 15 h: 3.95 ± 0.74			
						After 15 h: 6.73 ± 0.65		After 30 h: 8.03 ± 1.08			
						After 30 h: 7.35 ± 0.98		After 42 h: 9.96 ± 1.08			
								After 48 h: 11.81 ± 0.98			
						After 42 h: 7.49 ± 0.55	NAA (g%)	Time 0: 0.05 ± 0.006			
								After 9 h: 0.10 ± 0.08			
								After 15 h: 0.17 ± 0.03			
								After 30 h: 0.29 ± 0.07			
						After 48 h: 7.58 ± 0.63		After 42 h: 0.21 ± 0.08			
								After 48 h: 0.19 ± 0.06			
Hake (*Merluccius merluccius* L.)				-	pH	Time 0: 6.41 ± 0.37	TMA (mg%)	Time 0: 0.52 ± 0.03	-		
								After 9 h: 1.19 ± 0.04			
						After 9 h: 6.70 ± 1.12		After 15 h: 4.87 ± 1.12			
								After 30 h: 9.52 ± 0.89			
						After 15 h: 6.85 ± 0.74		After 42 h: 11.35 ± 0.88			
								After 48 h: 13.30 ± 1.56			
						After 30 h: 7.41 ± 0.56	NAA (g%)	Time 0: 0.04 ± 0.007			
								After 9 h: 0.14 ± 0.07			
						After 42 h: 7.61 ± 0.38		After 15 h: 0.27 ± 0.05			
								After 30 h: 0.42 ± 0.08			
						After 48 h: 7.73 ± 1.12		After 42 h: 0.31 ± 0.04			
								After 48 h: 0.21 ± 0.07			
Carp (*Cyprinus carpio*)	Czech Republic (aquaculture)	Filleted, packet on plastic bags and frozen (−20 °C)		Acceptable: 24 weeks. Cooked samples were scored between 0 (worst quality) to 100 (best quality); firmness, odor, color, and overall acceptability of frozen samples were scored between 0 (worst quality) to 5 (best quality). No absolute values are presented by the authors	Moisture (%)	Time 0: 71.8 ± 2.61	TBARS (µg MDA/kg)	Time 0: 0.03 ± 0.0	-		[48]
						Week 3: 70.9 ± 3.42					
						Week 8: 70.9 ± 2.31					
						Week 24: 69.8 ± 2.83					
					Fat content (%)	Time 0: 8.64 ± 2.36		Week 3: 0.06 ± 0.01			
						Week 3: 9.48 ± 3.23					
						Week 8: 11.8 ± 1.59					
						Week 24: 10.8 ± 3.28					
					Firmness (g): Significant decrease after 1 week of frozen storage						
								Week 8: 0.04 ± 0.01			
					Color: Frozen samples tended to be lighter and more yellow than the fresh ones.						
					pH	Time 0: 6.7 ± 0.12		Week 24: 0.04 ± 0.00			
						Week 3: 6.65 ± 0.10					
						Week 8: 6.65 ± 0.15					
						Week 24: 6.35 ± 0.24					
Cobia (*Rachycentron canadum*)	Vietnam (farmed)	Air blast freezer (−35 °C, 3 h) in polyethylene bags and stored 24 weeks	Not bleed	-	Water content	Day 0: 70.96 ± 0.31	TBARS: Not bleed samples with higher lipid oxidation products		-		[9]
						Day 12: 67.76 ± 0.28					
						Day 24: 61.30 ± 0.40					
					Total lipids	Day 0: 12.08 ± 0.12					
						Day 12: 8.60 ± 0.44					
						Day 24: 7.61 ± 0.74					
					Free fatty acids	Higher than bleed samples					
					Color	Lower L*^3^ and higher a*^3^ values than bleed samples					
			Throat cut and air bleed (15 min)		Water content	Day 0: 71.43 ± 0.33					
						Day 12: 67.87 ± 0.76					
						Day 24: 64.82 ± 0.98					
					Total lipids	Day 0: 12.71 ± 0.11					
						Day 12: 9.36 ± 1.53					
						Day 24: 8.03 ± 0.11					
			Throat cut and ice water bleed (4 ± 1 °C, 15 min)		Water content	Day 0: 70.87 ± 0.48					
						Day 12: 68.47 ± 0.73					
						Day 24: 68.41 ± 1.08					
					Total lipids	Day 0: 12.94 ± 0.06					
						Day 12: 10.12 ± 0.66					
						Day 24: 9.09 ± 0.59					

^1^—Nitrogen from amino acids. ^2^—Free fatty acids. ^3^—L*: lightness, a*: red/green value, b*: blue/yellow value.
